# Metabolic regulation of *Escherichia coli *and its *phoB *and *phoR *genes knockout mutants under phosphate and nitrogen limitations as well as at acidic condition

**DOI:** 10.1186/1475-2859-10-39

**Published:** 2011-05-20

**Authors:** Lolo Wal Marzan, Kazuyuki Shimizu

**Affiliations:** 1 Department of Bioscience & Bioinformatics, Kyushu Institute of Technology, Iizuka, Fukuoka 820-8502, Japan; 2 Institute of Advanced Bioscience, Keio University, Tsuruoka, Yamagata 997-0017, Japan

**Keywords:** *phoB *gene knockout, *phoR *gene knockout, phosphate limitation, pH, nitrogen limitation

## Abstract

**Background:**

The phosphorus compounds serve as major building blocks of many biomolecules, and have important roles in signal transduction. The phosphate is involved in many biochemical reactions by the transfer of phosphoryl groups. All living cells sophisticatedly regulate the phosphate uptake, and survive even under phosphate-limiting condition, and thus phosphate metabolism is closely related to the diverse metabolism including energy and central carbon metabolism. In particular, phosphorylation may play important roles in the metabolic regulation at acidic condition and nitrogen limiting condition, which typically appears at the late growth phase in the batch culture. Moreover, phosphate starvation is a relatively inexpensive means of gene induction in practice, and the *phoA *promoter has been used for overexpression of heterologous genes. A better understanding of phosphate regulation would allow for optimization of such processes.

**Results:**

The effect of phosphate (P) concentration on the metabolism in *Escherichia coli *was investigated in terms of fermentation characteristics and gene transcript levels for the aerobic continuous culture at the dilution rate of 0.2 h^-1^. The result indicates that the specific glucose consumption rate and the specific acetate production rate significantly increased, while the cell concentration decreased at low P concentration (10% of the M9 medium). The increase in the specific glucose uptake rate may be due to ATP demand caused by limited ATP production under P-limitation. The lower cell concentration was also caused by less ATP production. The less ATP production by H^+^-ATPase may have caused less cytochrome reaction affecting in quinone pool, and caused up-regulation of ArcA/B, which repressed TCA cycle genes and caused more acetate production. In the case of *phoB *mutant (and also *phoR *mutant), the fermentation characteristics were less affected by P-limitation as compared to the wild type where the PhoB regulated genes were down-regulated, while *phoR *and *phoU *changed little. The *phoR *gene knockout caused *phoB *gene to be down-regulated as well as PhoB regulated genes, while *phoU *and *phoM *changed little. The effect of pH together with lower P concentration on the metabolic regulation was also investigated. In accordance with up-regulation of *arcA *gene expression, the expressions of the TCA cycle genes such as *sdhC *and *mdh *were down-regulated at acidic condition. The gene expression of *rpoS *was up-regulated, and the expression of *gadA *was up-regulated at pH 6.0. In accordance with this, PhoB regulated genes were up-regulated in the wild type under P-rich and P-limited conditions at pH 6.0 as compared to those at pH 7.0. Moreover, the effect of nitrogen limitation on the metabolic regulation was investigated, where the result indicates that *phoB *gene was up-regulated, and PhoB regulated genes were also up-regulated under N-limitation, as well as nitrogen-regulated genes.

**Conclusion:**

The present result shows the complicated nature of the metabolic regulation for the fermentation characteristics upon phosphate limitation, acidic condition, and nitrogen limitation based on the transcript levels of selected genes. The result implies that the regulations under phosphate limitation, acidic condition, and nitrogen limitation, which occur typically at the late growth phase of the batch culture, are interconnected through RpoS and RpoD together with Pho genes.

## Background

The phosphorus compounds serve as major building blocks of many biomolecules, and have important roles in signal transduction [1]. The phosphate is contained in lipids, nucleic acids, proteins, and sugars, and is involved in many biochemical reactions by the transfer of phosphoryl groups [2]. Moreover, phosphate metabolism is closely related to the diverse metabolisms such as energy and central carbon metabolisms [3]. All living cells sophisticatedly regulate the phosphate uptake, and survive even under phosphate-limiting condition [4,5]. *Escherichia coli *contains about 15 mg of phosphate (P) per g (dry cell weight) [6]. Depending on the concentration of environmental phosphate, *E. coli *controls phosphate metabolism through Pho regulon, which forms a global regulatory circuit involved in a bacterial phosphate management [1,7]. The PhoR-PhoB two-component system plays an important role in detecting and responding to the changes of the environmental phosphate concentration [8-10]. It has been known that PhoR is an inner-membrane histidine kinase sensor protein that appears to respond to variations in periplasmic orthophosphate (P_i_) concentration through interaction with a phosphate transport system, and that PhoB is a response regulator that acts as a DNA-binding protein to activate or inhibit specific gene transcription [1,11-13]. The activation signal, a phosphate concentration below 4 μM, is transmitted by a phospho-relay from PhoR to PhoB. Phospho-PhoB in turn controls Pho regulon gene expressions. PhoB is phosphorylated by PhoR under phosphate starvation or by PhoM (or CreC) in the absence of functional PhoR [14-20].

The *E. coli *Pho regulon includes 31 (or more) genes arranged in eight separate operons such as *eda, phnCDEFGHIJKLMNOP, phoA, phoBR, phoE, phoH, psiE, pstSCAB-phoU*, and *ugpBAECQ *[21]. When P_i _is in excess, PhoR, Pst, and PhoU together turn off the Pho regulon, presumably by dephosphorylating PhoB. In addition, two P_i_-independent controls that may be form of cross regulation turn on the Pho regulon in the absence of PhoR. The sensor CreC, formerly called PhoM, phosphorylates PhoB in response to some (unknown) catabolite, while acetyl phosphate may directly phosphorylate PhoB [7]. When P_i _is in excess, P_i _is taken up by the low affinity P_i _transporter, Pit. Four proteins such as PstS, PstC, PstA and PstB form an ABC transporter important for the high-affinity capture of periplasmic inorganic phosphate (P_i_) and its low-velocity transport into the cytosol [22]. These proteins are encoded together with PhoU as the *pstSCAB-phoU *operon. PstS is a periplasmic protein that binds P_i _with high affinity. PstC and PstA are innermembrane channel proteins for P_i _entry, while PstB is an ATP-dependent permease that provides the energy necessary for P_i _transport from periplasm to cytosol. When phosphate is in excess, the Pst system forms a repression complex with PhoR, and prevents activation of PhoB. PhoU and PstB are also required for dephosphorylation of phospho-PhoB under P-rich condition [23]. Indeed, PhoU is essential for the repression of the Pho regulon under high phosphate condition [1]. It may be considered that PhoU acts by binding to PhoR, PhoB or PhoR/PhoB complex to promote dephosphorylation of phosphorylated PhoB or by inhibiting formation of the PhoR-PhoB complex [24].

It has been shown that *phoB *mutant does not synthesize alkaline phosphatase (*phoA *gene product) [25-30] and phosphate binding protein (*pstS *gene product) [26,29,30]. It was observed that *phoU *expression changed depending on phosphate concentration of the *phoB *mutant [31]. Since the *phoA *gene mutation leads to the decreased content of membrane proteins or completely lacks them, mutations in the *phoB *gene result in the loss of alkaline phosphate and two membrane proteins [32]. Nesmeianova et al. [25] found that *phoB *mutation leads to loss of polyphosphate kinase activity which catalyzes the synthesis of polyP in *E. coli*. Ault-Riché et al. [33] also found that the strains with deletion of *phoB *failed to accumulate polyP in response to osmotic stress or nitrogen limitation. Mutations in the *phoB *gene had no effect on *pepN *[34] and *lky *(*tolB*) expressions [35].

The expressions of the genes under the control of the PhoR-PhoB two-component system were found to be affected by the duration of P-limitation in response to phosphate starvation in *E. coli*. This means that the roles of the PhoR-PhoB two-component regulatory system seem to be more complex [10]. Although molecular level regulation by PhoR-PhoB under P-limitation has been investigated as stated above, little has been investigated about the effect of P- limitation on the overall metabolism and fermentation characteristics of *E. coli *so far. In the present study, therefore, we investigated the effect of phosphate limitation on the cell metabolism in *E. coli *in view of fermentation characteristics and gene transcript levels, since it is quite important for the development of microbial cell factories to understand the fermentation mechanism at the late growth phase in the batch culture, where nutrient starvation occurs. Moreover, the effect of *phoB *gene (and also *phoR *gene) knockout on the metabolism was also investigated under both P-rich and P-limited conditions to clarify the role of phosphate regulation. Since it has been implied that phosphate regulation is interconnected with acid tolerance and nitrogen regulation, we also investigated the effect of pH downshift and nitrogen limitation together with P-limitation on the metabolic regulation in *E. coli*, where those phenomena also occur at the late growth phase of the batch culture. Since phosphate starvation is a relatively inexpensive means of gene induction in practice, the *phoA *promoter has been used for overexpression of heterologous genes [36]. A better understanding of the Pho regulon would allow for optimization of such processes [22].

## Results

### Effect of phosphate limitation on the metabolism

In order to make clear the effect of phosphate limitation on the metabolism, aerobic continuous cultivation was conducted at the dilution rate of 0.2 h^-1 ^under different P concentrations. Additional file 1a shows the effect of P concentration on the fermentation characteristics of the wild type strain, where it indicates that the fermentation characteristics significantly changed when feed P concentration became low around 10% of the M9 medium. In particular, the specific glucose consumption rate and the specific acetate production rate became significantly higher, while cell concentration became significantly lower under such P-limiting condition. Table 1a also shows the detailed values.

**Table 1:** Fermentation characteristics of the wild type *E. coli *and its *phoB *and *phoR *mutants in the aerobic chemostat culture under different phosphate concentrations at the dilution rate of 0.2 h^-^^1 ^at pH 7.0.

Fermentation Parameters		P-rich (100%) condition	P-lower (55%) condition	P-limited (20%) condition	P-limited (15%) condition	P-limited (12.5%) condition	P-limited (10%) condition	P-limited (5%) condition	P-limited (1%) condition
Biomass concentration (g/l)	Wild	**3.86 ± 0.03**	**3.68 ± 0.05**	**3.47 ± 0.05**	**3.08 ± 0.02**	**2.78 ± 0.03**	**1.69 ± 0.03**	-	-
	
	Δ*phoB*	**3.44 ± 0.04**	**3.560 ± 0.011**	**-**	**-**	**-**	**3.64 ± 0.01**	**3.24 ± 0.02**	**0.050 ± 0.001**
	
	Δ*phoR*	**-**	**-**	**-**	**-**	**-**	**3.710 ± 0.112**	**-**	**-**

Glucose concentration (g/l)	Wild	**0.660 ± 0.004**	**0.760 ± 0.004**	**0.557 ± 0.001**	**0.700 ± 0.003**	**0.700 ± 0.003**	**1.85 ± 0.01**	**_**	**_**
	
	Δ*phoB*	**1.59 ± 0.29**	**1.66 ± 0.23**	**-**	**-**	**-**	**1.050 ± 0.001**	**1.330 ± 0.001**	**9.960 ± 0.001**
	
	Δ*phoR*	**-**	**-**	**-**	**-**	**-**	**0.910 ± 0.004**	**-**	**-**

Acetate concentration (g/l)	Wild	**0.046 ± 0.002**	**0.042 ± 0.001**	**0.410 ± 0.001**	**0.43 ± 0.07**	**0.41 ± 0.03**	**0.41 ± 0.02**	**_**	**_**
	
	Δ*phoB*	**0.255 ± 0.130**	**0.233 ± 0.030**	**_**	**_**	**_**	**0.346 ± 0.010**	**0.440 ± 0.010**	**0.002 ± 0.002**
	
	Δ*phoR*	**-**	**-**	**-**	**-**	**-**	**0.0035 ± 0.0010**	**-**	**-**

Specific glucose uptake rate (mmol/gDCW/h)	Wild	**2.69 ± 0.05**	**2.79 ± 0.02**	**3.024 ± 0.005**	**3.350 ± 0.001**	**3.717 ± 0.002**	**5.36 ± 0.01**	**_**	**_**
	
	Δ*phoB*	**2.72 ± 0.09**	**2.60 ± 0.07**	**_**	**_**	**_**	**2.730 ± 0.003**	**2.970 ± 0.001**	**0.890 ± 0.001**
	
	Δ*phoR*	**-**	**-**	**-**	**-**	**-**	**2.70 ± 0.004**	**-**	**-**

Specific acetate production rate (mmol/gDCW/h)	Wild	**0.040 ± 0.002**	**0.0380 ± 0.0001**	**0.394 ± 0.010**	**0.465 ± 0.003**	**0.491 ± 0.030**	**0.81 ± 0.02**	**_**	**_**
	
	Δ*phoB*	**0.247 ± 0.080**	**0.218 ± 0.033**	**_**	**_**	**_**	**0.317 ± 0.001**	**0.452 ± 0.010**	**0.133 ± 0.002**
	
	Δ*phoR*	**-**	**-**	**-**	**-**	**-**	**0.0031 ± 0.0010**	**-**	**-**

Note: " - " indicates that no data was collected for this condition. The standard deviation was obtained by triplicate measurements.

**Figure 1:**
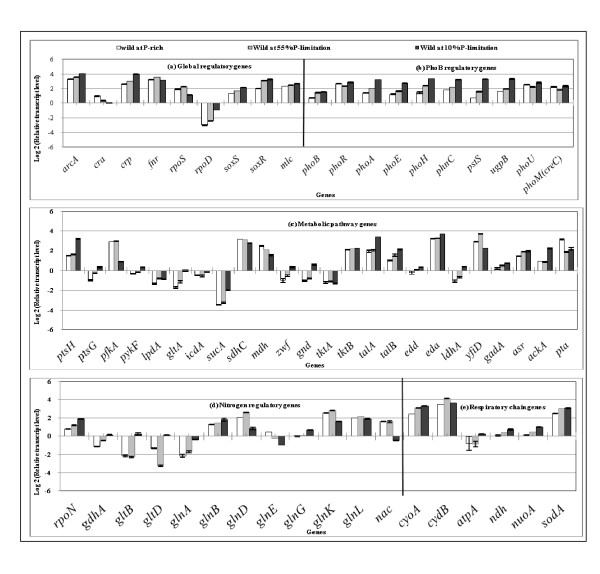
**Comparison of the transcript levels of the wild type *E. coli *cultivated with different P concentrations of the feed (100%, 55%, 10%): (a) global regulatory genes, (b) PhoB regulatory genes, (c) metabolic pathway genes, (d) nitrogen regulatory genes, and (e) respiratory chain genes**.

Figure 1 shows the effect of P concentration on the transcript levels, where Figure 1b indicates that *phoB *transcript level increased as P concentration decreases, and *phoB *regulated genes such as *phoA, phoE, phoH, phnC, pstS*, and *ugpB *were all increased in a similar fashion, and *eda *transcript level also changed in a similar fashion (Figure 1c). Note that *phoU *and *phoM *changed in a similar fashion as *phoR*, and also that the transcript level of *rpoD*, which encodes the RNA polymerase holoenzyme containing σ^70^, increased in a similar fashion as PhoB regulatory genes [37]. Figure 1a also indicates that the transcript level of *arcA *increased as P concentration decreases, and those of *sdhC *and *mdh *genes decreased (Figure 1c and Additional file 2). Figure 1 also shows that *cra *transcript level decreased, and thus the transcript levels of *ptsH *and *pykF *increased (Additional file 2). Those are consistent with the increased specific glucose consumption rate (Table 1 and Additional file 1a). The decrease in *cra *transcript level may be due to higher glucose concentration. The transcript level of *fnr *is somewhat different from *arcA *but that of *yfiD *changed in a similar fashion as *fnr *(Additional file 2). Moreover, Figure 1a also indicates that *soxR/S *transcript levels increased as P concentration decreases, and accordingly the transcript levels of *rpoD, zwf *and *sodA *changed in a similar fashion (Additional file 2). The respiratory chain genes such as *atpA, ndh*, and *nuoA *also changed in a similar fashion, implying that the respiration is activated under P-limitation. Figure 1d shows that *rpoN *which encode σ^54 ^increases as P concentration decreased.

**Figure 2:**
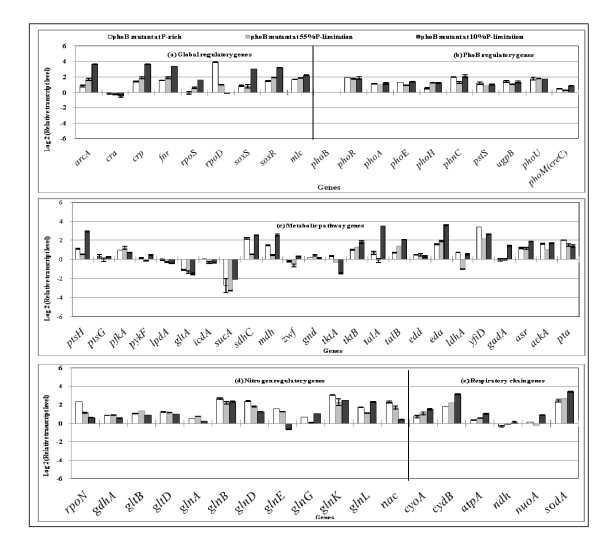
**Comparison of the transcript levels of the *phoB *mutant *E. coli *cultivated with different P concentrations of the feed (100%, 55%, 10%): (a) global regulatory genes, (b) PhoB regulatory genes, (c) metabolic pathway genes, (d) nitrogen regulatory, and (e) respiratory chain genes**.

Table 1 also shows the effect of *phoB *gene knockout on the fermentation characteristics under both P-rich and lower P conditions, where it indicates that the glucose concentration increased and cell concentration decreased for the *phoB *mutant as compared to the wild type, and that the specific acetate production rate was higher at P-rich condition and 55% of P concentration for the *phoB *mutant as compared to the wild type. It is surprising that the fermentation characteristics were less affected even under P-limitation (10% and 5%) for the *phoB *mutant, whereas the wild type shows significant changes at 10% of P concentration. In the case of *phoB *mutant, cell could survive even at 1% of P concentration (Table 1 and Additional file 1b). Figure 2 indicates that the transcript levels of PhoB regulated genes such as *phoA, phoE, phoH, pstS, ugpB *and *phoM *were down-regulated, whereas *phoR *and *phoU *changed little, as compared to those of wild type. In a similar fashion as the wild type, the transcript level of *arcA *increased while *cra *decreased as P concentration decreased for the *phoB *mutant, which implies that those phenomena are *phoB *independent. The transcript levels of *soxR *and *rpoS *increased and *sodA *as well as respiratory chain genes such as *cyoA*, *ndh *and *nuoA *increased in a similar fashion as P concentration decreased for the *phoB *mutant, which implies that the activation of the respiratory chain is *phoB*-independent, but P-concentration dependent.

**Figure 3:**
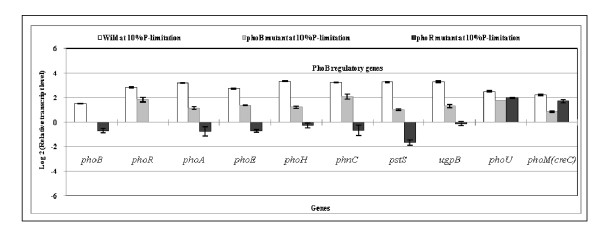
**Comparison of the transcript levels of Pho regulon genes for the wild type, *phoB *and *phoR *mutants cultivated at 10% P- concentration**.

In order to confirm the result for *phoB *mutant, the effect of *phoR *gene knockout on fermentation characteristics and some selected gene transcript levels were also investigated for the case of 10% of P concentration as given in Table 1 and Figure 3. Table 1 indicates that the cell concentration and the glucose concentration for *phoR *mutant are similar to those of *phoB *mutant, whereas acetate concentration became quite low at P-limiting condition for the *phoR *mutant. Figure 3 indicates that the *phoB *regulated genes such as *phoA, phoE, phoH, phnC, pstS, ugpB *were more down-regulated for the *phoR *mutant as compared to *phoB *mutant, whereas *phoU *and *phoM *(*creC*) were less affected by *phoR *gene knockout.

### Effect of culture pH and phosphate limitation on the metabolism

**Figure 4:**
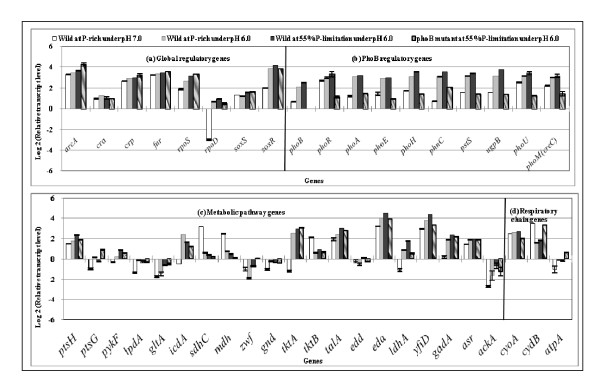
**Comparison of the transcript levels of the wild type and its *phoB *mutant cultivated under different pH condition and different P concentrations: (a) global regulatory genes, (b) PhoB regulated genes, (c) metabolic pathway genes, and (d) respiratory chain genes**.

Table 2 shows the effect of pH and phosphate limitation on the fermentation characteristics in the continuous culture of *E. coli *at the dilution rate of 0.2 h^-1^, where it indicates that more acetate was formed with higher glucose uptake rate, while the cell concentration became lower at pH 6.0 as compared to the case of pH 7.0. Note that the fermentation characteristics were different even between 100% and 55% of phosphate concentration under lower pH value. Figure 4 shows the effect of culture pH on the transcript levels, where it indicates that *arcA *gene was up-regulated (P < 0.01), and the TCA cycle genes such as *sdhC *and *mdh *were down-regulated accordingly (P < 0.01 and P < 0.01, respectively). Note that *icdA *gene was up-regulated (P < 0.01), which coincided with the up-regulation of *cra *gene (P < 0.01) (Additional file 2). Figure 4 also shows that the transcript level of *rpoS *was up-regulated, and the expression of *gadA *(glutamate decarboxylase) gene was up-regulated at pH 6.0 [38]. The *yfiD *gene, which encodes acid-inducible protein [39], was also up-regulated at lower pH. Figure 4 also shows that *phoB *gene was up-regulated and the PhoB regulated genes such as *phoA, phoE, phoH, phnC, pstS*, and *ugpB *as well as *phoR*, *phoU, phoM *and *eda *were up-regulated (P < 0.01 for all genes). This means that acid stress and phosphate regulation are directly or indirectly interconnected [4].

**Table 2:** Fermentation characteristics of the wild type *E. coli *and its *phoB *mutant in the aerobic chemostat culture under two phosphate concentrations (100% and 55%) and two pH values (7.0 and 6.0) at the dilution rate of 0.2 h^-^^1^.

Fermentation parameters		P-rich condition (100%)		Lower P concentration (55%)
		
		pH 7.0	pH 6.0	pH 6.0
Biomass concentration (g/l)	Wild	**3.86 ± 0.03**	**3.680 ± 0.002**	**1.960 ± 0.001**
	
	Δ*phoB*	**3.44 ± 0.04**	**_**	**3.040 ± 0.001**

Glucose concentration (g/l)	Wild	**0.660 ± 0.004**	**1.053 ± 0.010**	**0.677 ± 0.050**
	
	Δ*phoB*	**1.59 ± 0.29**	**_**	**1.145 ± 0.020**

Acetate concentration (g/l)	Wild	**0.046 ± 0.002**	**0.468 ± 0.003**	**0.519 ± 0.001**
	
	Δ*phoB*	**0.255 ± 0.130**	**_**	**0.510 ± 0.007**

Specific glucose uptake rate (mmol/gDCW/h)	Wild	**2.69 ± 0.05**	**2.700 ± 0.001**	**5.285 ± 0.031**
	
	Δ*phoB*	**2.72 ± 0.09**	**_**	**3.24 ± 0.01**

Specific acetate production rate (mmol/gDCW/h)	Wild	**0.040 ± 0.002**	**0.424 ± 0.003**	**0.882 ± 0.001**
	
	Δ*phoB*	**0.247 ± 0.080**	**_**	**0.559 ± 0.007**

Note: " - " indicates that no data was collected for this condition. The standard deviation was obtained by triplicate measurements.

Figure 4 also shows the effects of lower pH and lower P-concentration on the transcript levels (3^rd ^bars), where *phoB *gene was further up-regulated, and the Pho regulon genes such as *phoR, phoH, phnC, pstS, ugpB*, and *phoU *were all further up-regulated at lower P concentration at pH 6.0. The *rpoS *gene further increased (P < 0.01), and *gadA *gene was also further up-regulated (P < 0.01). Figure 4 shows that *arcA *transcript level tended to be up-regulated though not significant, and this may have caused down-regulations of *sdhC *and *mdh *(P < 0.01 and P < 0.01, respectively) under P-limitation as compared to P-rich condition at acidic condition.

Table 2 shows the effects of lowering pH and P concentration on the fermentation characteristics of *phoB *gene knockout mutant as well, where it indicates that the glucose concentration increased for the *phoB *mutant as compared to the wild type under both pH 7.0 and 6.0. Figure 4 shows the comparison of the transcript levels between the wild type (3^rd ^bars) and its *phoB *mutant (4^th ^bars) at pH 6.0, where it indicates that the *phoB *regulated genes such as *phoA, phoE, phoH, phnC, pstS, ugpB *(P < 0.01 for all genes) as well as *phoR*, *phoU, phoM *and *eda *were all significantly down-regulated (P < 0.01 for all genes) for the *phoB *mutant. The transcript levels of TCA cycle genes such as *sdhC *and *mdh *were down-regulated for the *phoB *mutant as compared to the wild type.

### Effect of nitrogen limitation

**Figure 5:**
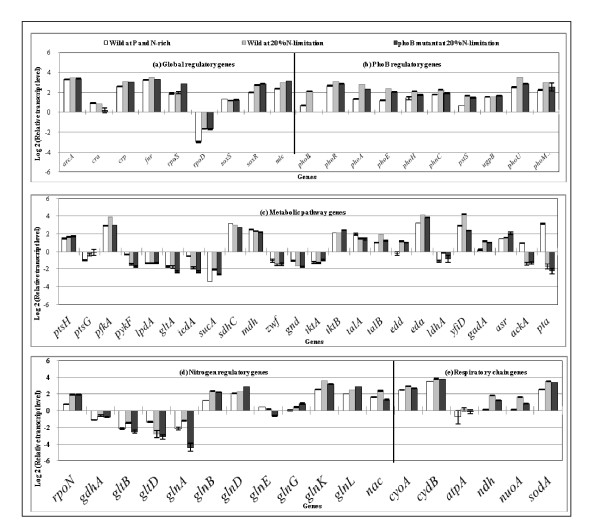
**Comparison of the transcript levels of the wild type cultivated at 100% and 20% N-limitation and *phoB *mutant cultivated at 20% N-limitation: (a) global regulatory genes, (b) PhoB regulatory genes, (c) nitrogen regulatory genes, (d) metabolic pathway genes, and (e) respiratory chain genes**.

Table 3 shows the effect of nitrogen (N) limitation and lower P concentration on the fermentation characteristics, where the specific glucose consumption rate and the specific acetate production rate increased, while cell concentration decreased for the case of N-limitation as compared to N-rich condition. Those changes were further enhanced at lower P concentration. Figure 5 shows the effect of N-limitation on the transcript levels of several genes, where it indicates that *rpoN *transcript level increased and *glnA, L*, *G*, *gltB*, *glnD, glnK *and *nac *genes were up-regulated (P < 0.01 for all genes), while *glnE *gene was down-regulated (P < 0.01). Figure 5 also shows that the transcript level of *phoB *gene was up-regulated and PhoB regulated genes such as *phoA, phoE, phoH, phnC, pstS *were increased (P < 0.01 for all genes) as well as *phoR, phoU*, and *phoM *(P < 0.01 for all genes) under N-limitation as compared to N-rich condition. The TCA cycle genes such as *sdhC *and *mdh *decreased (P < 0.01 for both genes), which may have caused TCA cycle to be repressed, which corresponds to the increase in the specific acetate production rate. The *ptsG*, *ptsH *and *pfkA *gene expressions increased (P < 0.01 for all genes), which corresponds to the increase in the specific glucose consumption rate. The transcript levels of *fnr *and *yfiD *were up-regulated (P < 0.01 for both genes) in a similar fashion. Moreover *soxR *increased and the respiratory chain genes such as *cyoA, cydB*, *ndh *and *nuoA *as well as *sodA *were all up-regulated under N-limitation (P < 0.01 for all genes).

**Table 3:** Fermentation characteristics of the wild type *E. coli *and its *phoB *mutant in the aerobic chemostat culture under different nitrogen and phosphate concentrations at the dilution rate of 0.2 h^-^^1 ^at pH 7.0.

Fermentation parameters		N and P-rich condition (100%)	N -limited (20%) and P-rich condition	N-limited (20%) and lower P concentration (55%)
Biomass concentration (g/l)	Wild	**3.86 ± 0.03**	**1.753 ± 0.005**	**1.68 ± 0.01**
	
	Δ*phoB*	**3.44 ± 0.04**	**1.703 ± 0.005**	**1.59 ± 0.01**

Glucose concentration (g/l)	Wild	**0.660 ± 0.004**	**5.39 ± 0.01**	**4.10 ± 0.01**
	
	Δ*phoB*	**1.59 ± 0.29**	**2.890 ± 0.006**	**4.81 ± 0.01**

Acetate concentration (g/l)	Wild	**0.046 ± 0.002**	**0.486 ± 0.002**	**0.502 ± 0.004**
	
	Δ*phoB*	**0.255 ± 0.130**	**0.475 ± 0.004**	**0.495 ± 0.010**

Specific glucose uptake rate (mmol/gDCW/h)	Wild	**2.69 ± 0.05**	**2.92 ± 0.01**	**3.90 ± 0.01**
	
	Δ*phoB*	**2.72 ± 0.09**	**4.64 ± 0.01**	**3.63 ± 0.02**

Specific acetate production rate (mmol/gDCW/h)	Wild	**0.040 ± 0.002**	**0.923 ± 0.010**	**0.995 ± 0.010**
	
	Δ*phoB*	**0.247 ± 0.080**	**0.928 ± 0.010**	**1.037 ± 0.020**

Note: The standard deviation was obtained by triplicate measurements.

Table 3 also shows the effect of lower P concentration on the fermentation characteristics of *phoB *mutant under N-limitation, where it indicates that the cell concentration decreased, while acetate and glucose concentrations increased under N-limitation as compared to N-rich condition for the *phoB *mutant. Figure 5 indicates (by comparison of the 2^nd ^and 3^rd ^bars) that phoB regulated genes such as *phoA, phoE, phnC, pstS*, *phoR, phoU*, (P < 0.01 for all genes) as well as *phoH *and *phoM *were down-regulated (P < 0.05 for the two genes) as compared to the wild type. Although *rpoN *transcript level changed little, such genes as *glnD, glnG, glnL *were up-regulated (P < 0.01 for all genes), whereas *gltD *(P < 0.5), and *glnA, glnE, glnK, nac *genes were down-regulated (P < 0.01 for all genes).

## Discussion

It was shown that the glycolysis was activated under phosphate limiting condition by the data of the specific growth rate (Table 1) and by the corresponding gene transcript levels (Figure 1). This may be due to ATP demand caused by the decrease in ATP formation with limited amount of available phosphate as implied by Kobeman et al. [40] who investigated the effect of [ATP]/[ADP] ratio on the glycolytic flux. The lower cell concentration under P-limitation may also be due to lower ATP formation as we investigated previously on the relationship between the cell growth rate and the specific ATP production rate [41,42].

Moreover, phosphate limitation causes less ATP production by H^+^-ATPase, which causes less quinol oxidation by Cyo, which in turn affected quinone pool size, and thus activate ArcA/B, which represses the TCA cycle genes, and in turn produced more acetate. The similar situation has also been seen in *cyoA *and *cydB *genes knockout mutants in our previous investigation [43].

Figure 1b indicates that *phoB *gene transcript level increased as P concentration decreases in the wild type, and Figure 1a indicates that *rpoD *also increased as P concentration decreases. The *phoA, phoE, phoH, phnC, pstS*, and *ugpB *were all increased in a similar fashion as that of *rpoD *as mentioned in the result section. Figure 1a indicates that the expression pattern of *rpoS *is somewhat different. When cells enter into P_i_-starvation phase in the batch culture, the Pho regulon is activated, and σ^S ^starts to accumulate in the cytosol [1,44,45]. The promoters of the Pho genes are recognized by σ^D ^- associated RNA polymerase. A mutation in *rpoS*, significantly increases the level of AP (Alkaline phosphatase) activity, and the overexpression of σ^S ^inhibits it [46]. It has been reported that in *rpoS *mutant, the expression of AP was considerably higher than in wild-type strain, implying that σ^S ^is involved in the regulation of AP. Other Pho genes such as *phoE *and *ugpB *are likewise affected by σ^S^. The *rpoS *may inhibit the transcriptions of *phoA, phoB, phoE*, and *ugpB*, but not that of *pstS *[46]. Figure 1ab indicates that Pho genes are highly expressed as compared to low *rpoS *transcript level in the case of P-limitation. In contrast, *pst *may be transcribed by both σ^S ^and σ^D^. The Pho regulon is thus evolved to maintain a trade-off between cell nutrition and cell survival during P_i_-starvation [46]. The previous reports suggest that the Pho regulon and the stress response are interrelated [45-50].

*E. coli *cells have been demonstrated to exhibit acid resistance by such genes as *gadAB *which encode glutamate decarboxylase and *gadC *which encodes glutamate: γ-amino butyric acid (GABA) antiporter. Glutamate decarboxylase production has been shown to increase in response to acid, osmotic and stationary phase signals [51,52]. In the typical batch culture, organic acids are most accumulated at the late growth phase or the stationary phase. It was shown that *gadA *was PhoB-dependently up-regulated in the present study (Figure 4), and this indicates that this gene is indirectly regulated by PhoB. Note that Figure 4 indicates that *gadA *gene expression decreased for the *phoB *mutant under acidic condition (4^th ^bar), while *rpoS *increased. This suggests that phosphate starvation and acid stress responses may be interconnected [4].

Figure 4 also indicates that *yfiD *transcript level increased at acidic condition. It has been shown that the expression of *yfiD *gene is induced at acidic condition, and this reduces the accumulation of acidic metabolite and products [39]. The anaerobic transcription factor Fnr (Fumarate and nitrate reduction regulator) has been shown to be the major regulator of *yfiD *expression, and ArcA was shown to enhance anaerobic *yfiD *expression [39]. Figure 4 indicates that *yfiD *transcript level changed in a similar fashion as *rpoD *rather than *arcA *and *fnr*. It has been known that the transcriptional regulator Fnr of *E. coli *functions as an O_2 _sensor, and the protein is in the active form and predominately exists as a homo-dimer with one [4Fe-4S] cluster per monomer under anoxic conditions. In the presence of oxygen, [4Fe-4S] FNR is converted to [2Fe-2S] FNR cluster and finally to apoFnr, which is no longer active in gene regulation [53,54]. Nevertheless, *fnr *gene transcript level changed, which indicates that Fnr does not play its conventional role, and may have some role under aerobiosis, but it is not clear at this stage. Figure 4 also indicates that *soxR *increased at acidic condition. The acidic condition may affect membrane properties such as lipid content, thus effectively changing the proton permeability. The increased expression of *soxR *regulates the removal of damaging oxidizing agents [55].

As expected, the acid inducible *asr *gene transcript level increased at pH 6.0 as compared to the case at pH 7.0 as shown in Figure 4c. The *asr *gene has been reported to be under the transcriptional control of the PhoR/PhoB two component system in *E. coli *[56]. Figure 1c indicates that *asr *gene transcript level increased as P concentration decreases in accordance with the change in *phoB *transcript level. Asr is thought to play a role similar to that of the *E. coli *periplasmic protein HdeA, which serves as a proton sink or a chaperone for protecting periplasmic proteins from the deleterious effects at lower pH [57]. As another example, the PhoR/PhoB system has been suggested to sense external acidity and regulate the transcription of genes that are important for acid shock resistance [56,58-61].

The presence of glucose or mutations in *cya *or cAMP receptor protein (*crp*) gene leads to induction of *phoA *gene in *phoR *mutant. This induction requires the sensor PhoM (CreC) and the regulator PhoB [62]. However, PhoM (CreC) may not detect glucose per se, where it may detect an intermediate in the central metabolism. Therefore, *cya *or *crp *mutation may indirectly affect PhoM (CreC) - dependent control. In addition to P_i _control, two P_i_-independent controls may lead to activation of PhoB. These two may be connected to control pathways in carbon and energy metabolisms, in which intracellular P_i _is incorporated into ATP. One P_i _independent control is the regulation by the synthesis of AcP, where P_i _is incorporated into ATP at Ack (acetate kinase) pathway. AcP may act indirectly on PhoB.

In *E. coli*, assimilation of N-source such as NH_4_^+ ^using α-KG results in the synthesis of glutamate and glutamine. Glutamine synthetase (GS encoded by *glnA*) catalyzes the only pathway for glutamine biosynthesis. Glutamate can be synthesized by two pathways through combined actions of GS and glutamate synthase (GOGAT encoded by *gltBD*) forming GS/GOGAT cycle, or by glutamate dehydrogenase (GDH encoded by *gdhA*). Under N-limitation, ammonium enters into the cell via AmtB and is converted to Gln by GS, and UTase (encoded by *glnE*) uridylylates both GlnK (encoded by *glnK*) and GlnB (encoded by *glnB*) [63]. Figure 5d (1^st ^and 2^nd ^bars) indicates that *rpoN *increased under N-limitation, and *glnALG, glnB, glnK *as well as *nac *genes increased as stated above. On the other hand, under N-rich condition, UTase deurydylylates GlnK and GlnB. GlnK complexes with AmtB, thereby inhibiting the transporter via AmtB, where GlnB interacts with NtrB (encoded by *glnL*) and activates its phosphatase activity leading to dephosphorylation of NtrC (encoded by g*lnG*), and NtrC- dependent gene expression ceases [63], thus the nitrogen regulation is affected by the phosphorylation caused by the available P source.

Figure 5d (2^nd ^and 3^rd ^bars) indicates that *glnB *and *glnK *transcript levels decreased, and *glnA *transcript level became lower under P-limitation as compared to P-rich condition under N-limitation. In the case under N-limitation, C/N ratio increases where α-KG is withdrawn via GDH, which affects the TCA cycle flux. A decreased flow through the TCA cycle would be expected to cause an increase in AcCoA pool and caused more acetate overflow.

Although little research has been done, it is quite important in practice to analyze the metabolism at the late growth phase and the stationary phase in the batch culture, where the medium is nutrient poor indicating carbon, phosphorous, and nitrogen limitations, as well as lower pH. When a particular nutrient becomes limiting, the first response is scavenging. These scavenging regulons include cAMP-Crp which allows for the use of alternative carbon sources such as acetate, and the two-component regulatory systems PhoR/PhoB and NtrB/NtrC, which control scavenging for phosphorus and nitrogen, respectively. Both Crp and Ntr systems survey nutrient status through intracellular metabolites, where Crp recognizes cAMP, while NtrC responds to glutamine. The Pho system, on the other hand, monitors inorganic phosphate levels via the activity of the Pst transport system [1]. The sigma factor responsible for the general stress resistance is RpoS (σ^38^) upon starvation. Note that the housekeeping sigma factor RpoD (σ^70^) is homologous to RpoS. Carbon starvation is one of the strongest inducers of RpoS, where regulation of RpoS occurs at the level of proteolysis by C1pXP. This regulation is made by *sprE*, which encodes a response regulator SprE (also called RssB) [64]. RpoS plays also an important role under phosphate limiting condition. However, its regulation mechanism is different. Note that while carbon starvation completely shuts down the central metabolism, it continues upon phosphate starvation [65]. In contrast to carbon and nitrogen starvation, the PhoR/PhoB two component system, either directly or indirectly regulates the translation of *rpoS *mRNA [45]. Since PhoB is a transcriptional regulator, its effects on *rpoS *translation may be indirect, where small noncoding RNAs (sRNAs) are important regulators of translation of mRNA. The sRNAs require the RNA chaperone Hfq for the formation of the RNA-RNA duplex, and there are several RNAs known to affect *rpoS *translation [66]. Namely, impeding phosphorous starvation is sensed as diminished activity of the Pst transporter, which causes autophosphorylation of PhoR, which then phosphorylate PhoB. The phosphorylated PhoB directly or indirectly activates transcription of an sRNA that stimulates translation of *rpoS *mRNA, thus elevating levels of RpoS [66]. Note that the regulation may be more complicated, since Figure 2a indicates that *rpoS *level increased as P concentration decreases even for the *phoB *mutant as also noted by Peterson et al. [66].

Upon nitrogen starvation, ppGpp levels were known to increase, and there might be some correlations between levels of ppGpp and RpoS levels. RpoS is not stabilized upon nitrogen starvation like it is upon carbon starvation or phosphate starvation, and thus the regulation mechanism may be different, suggesting an increase in the activity of RpoS. [66]. Similar proteins are induced following starvation for carbon, phosphorous and nitrogen [67,68], where RpoS-dependent genes are induced upon starvation. Although the activity of RpoS seems to be critical for nitrogen starvation, there are many players that affect the competition between RpoS and RpoD, including Rsd, 6S RNA, and ppGpp [66]. The role that the NtrB/C nitrogen scavenging system plays in regulating RpoS is unclear.

**Figure 6:**
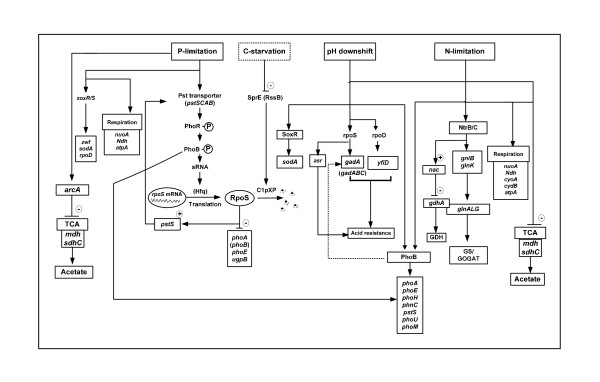
**Schematic illustrations for the metabolic regulation mechanism**.

The overall regulation mechanism may be illustrated schematically as Figure 6.

Similar mechanism might exist in *phoB *mutant *E. coli*, and further investigation is needed to clarify this.

Finally, it seems to be surprising that *phoB *(and also *phoR*) mutant could survive even under strict P limiting condition as compared to wild type as shown in Table 1 and Additional file 1. Figure 2 indicates that Pho regulon genes were insensitive to P concentration as expected, whereas global regulatory genes (Figure 2a), metabolic pathway genes (Figure 2c), and respiratory chain genes (Figure 2e) changed significantly. It has recently been reported that *phoB *mutant was more sensitive to hydrogen peroxide, but that *phoB *mutant was more resistant to high osmolarity and acid conditions compared to the wild type of *Vibrio cholerae *[69].

## Conclusion

The present investigation clarified the effect of phosphate limitation, nitrogen limitation, and acidic condition on the metabolism in view of gene transcript levels. Moreover, the present study implies that the metabolic regulations under phosphate limitation, nitrogen limitation and acidic condition are interconnected. These phenomena occur at the late growth phase in the batch culture. The present result is useful for the analysis of the metabolism changes during late growth phase and/or stationary phase.

## Materials and methods

### Bacterial strains used and culture conditions

The strains used in the present study were *Escherichia coli *BW25113 (*lacI**^q ^***rrnB**_T14 _**ΔlacZ**_wJ16 _***hsdR514 *ΔaraBAD**_AH33 _**Δ*rhaBAD ***_LD78_**), its *phoB *gene knockout mutant (JW0389) and *phoR *mutant (JW0390). The mutants were constructed by one-step inactivation of chromosomal *phoB *and *phoR *genes, respectively [70]. Continuous cultivations were carried out in a 2-L fermentor (M-100, Tokyo, Rikakiki Co., Tokyo, Japan), where the temperature was kept constant at 37°C. The pH of the broth was maintained either at 7.0 ± 0.1 or 6.0 ± 0.1 with a pH controller by automatic addition of 2.0 M HCl or 2.0 M NaOH. The aerobic condition was ascertained by controlling the stirring speed at 350 rpm with the constant air flow rate of 1 L min^-1^, which has been shown to be 30-40% of air saturation. The CO_2 _and O_2 _concentrations were monitored using an off-gas analyzer (BMJ-02 PI, ABLE Co., Japan).

The M9 minimal medium was used where it contained 10 g of glucose per liter, 48 mM Na_2_HPO_4,_, 22 mM KH_2_PO_4_, 10 mM NaCl, and 30 mM (NH_4_)_2_SO_4_. The following components were filter sterilized and then added (per liter of final medium): 1 ml of 1 M MgSO_4_, 1 ml of 0.1 mM CaCl_2_, 1 ml of 1 mg of vitamin B1 per liter, and 10 ml of trace element solution containing (per liter) 0.55 g of CaCl_2_.2H_2_O, 1.67 g of FeCl_3_, 0.10 g of MnCl_2_.4H_2_O, 0.17 g of ZnCl_2_, 0.043 g of CuCl_2_.2H_2_O, 0.06 g of CoCl_2_.6H_2_O and 0.06 g Na_2_MoO_4_.2H_2_O. Continuous cultivations were performed at the dilution rate of 0.2 h^-1^, where the feed glucose concentration was 10 g/L, and several different phosphate concentrations were considered: P-rich or 100% P concentration (2.99 g/L of KH_2_PO_4 _and 6.81 g/L of Na_2_HPO_4_) and P-limitation or 10% of P concentration (0.229 g/L of KH_2_PO_4 _and 0.681 g/L of Na_2_HPO_4_). Several other P concentrations in between were also investigated. For the nitrogen limited condition, 6.0 mM of (NH_4_)_2_SO_4 _was used instead of 30.0 mM. The continuous (chemostate) culture was controlled by adjusting the rotation speed of input and output pumps of the fermentor, where the rotation speed of output pump was adjusted to keep the broth volume constant, while the rotation speed of input pump was adjusted to set the dilution rate. In the present investigation, the dilution rate was set at 0.2 h^-1^, where glucose limitation was not observed at such dilution rate. If the dilution rate was decreased less than about 0.1 h^-1^, the glucose concentration becomes undetectable level, and both glucose and phosphate limitation may occur, and the changes of the transcript levels may be direct or indirect. To avoid such situation, we set the dilution rate to be at 0.2 h^-1^. The triplicate samples were taken after 4-5 residence times where the steady state was ascertained.

### Measurements of biomass and extracellular metabolite concentrations

Cell concentration was measured by the optical density (OD) of the culture broth at 600 nm wave length with a spectrophotometer (Ubet-30, Jasco Co., Tokyo, Japan), and then converted to dry cell weight (DCW) per liter based on the relationship between OD_600nm _and DCW previously obtained [71]. Glucose concentration was measured using enzymatic kit (Wako Co., Osaka, Japan). Acetate and lactate concentrations were also measured using enzymatic kits (Boehringer Co., Mannheim, Germany). Triplicate measurements were made for each sample to compute the standard deviation.

### RNA preparation, design of PCR primers

Total RNA was isolated from *E. coli *cells by Qiagen RNeasy Mini Kit (QIAGEN K.K., Japan) according to the manufacturer's recommendation. The quantity and purity of the RNA were determined by the optical density measurements at 260 and 280 nm and by 1% formaldehyde agarose gel electrophoresis. Criteria for the design of the gene-specific primer pairs were followed according to Sambrook and Russel [72]. The primers used in this study were described elsewhere [73,74], except those as given in additional file 3. The primers used in this study were synthesized at Hokkaido System Science Co. (Sapporo, Hokkaido Japan). In all cases, the primer-supplied company confirmed the absolute specificity of the primers.

### cDNA synthesis and PCR amplification

RT-PCR reactions were carried out in a TaKaRa PCR Thermal Cycler (TaKaRa TP240, Japan) using Qiagen One Step RT-PCR Kit (QIAGEN K.K., Japan). The reaction mixture was incubated for 30 min at 50°C for reverse transcription (cDNA synthesis) followed by 15 min incubation at 95°C for initial PCR activation. Then the process was subjected to 30 cycles of amplification which consisted of a denaturing step (94°C for 1 min), annealing step (approximately 5°C below melting temperature, of primers for 1 min), and an extension step (72°C for 1 min), and finally the reaction mixture of 25 μl was subjected for 10 min at 72°C for final extension. To check for nucleic acid contamination, one negative control was run in every round of RT-PCR. This control lacks the template RNA in order to detect possible contamination of the reaction components. 5 μl of amplified products were run on 1.8% agarose gel. Gels were stained with 1 mg ml^-1 ^of ethidium bromide, photographed using a Digital Image Stocker (DS-30, FAS III, Toyobo, Osaka, Japan) under UV light and analyzed using Gel-Pro Analyzer 3.1 (Toyobo, Osaka, Japan) software. In order to determine the optimal amount of input RNA, the two-fold diluted template RNA was amplified in RT-PCR assay under identical reaction condition to construct a standard curve for each gene product. When the optimal amount of input RNA was determined for each gene product, RT-PCR was carried out under identical reaction condition to detect differential transcript levels of genes. The gene *dnaA*, which encodes *E. coli *DNA polymerase and is not subjected to variable expression, i.e. abundant expression at relatively constant rate in most cells, was used as an internal control in the RT-PCR determinations. The gene expressions are given as relative values to that of *dnaA*. The selection of genes was made based on global regulator-metabolic pathway gene relationships (Additional file 2).

To calculate the standard deviation, RT-PCR was independently performed three times under identical reaction condition. To ensure that the observed expression changes were statistically significant, the Student's t-test was applied.

## Abbreviations

α-KG: 2-Ketoglutarate; AcCoA: Acetyl-Coenzyme A; Ack: Acetate kinase; AcP: Acetyl Phosphate; AmtB: Ammonium transport protein; AP: Alkaline phosphatase; ATP: Adenosine Tri-Phosphate; cAMP: Cyclic adenosine monophosphate; cAMP-Crp: cAMP receptor protein; DCW: Dry Cell Weight; [4Fe-4S]: In a number of iron-sulfur proteins; GABA: α-Aminobutyric acid; GDH: Glutamate Dehydrogenase; Gln: Glutamine; GOGAT: Glutamate Syntheses; GS: Glutamine Synthetase; HdeA: Periplasmic protein; N: Nitrogen; OAA: Oxaloacetic Acid; P: Phosphate; PEP: Phospho-enol-pyruvate; ppGpp: Guanosine tetraphosphate; PYR: Pyruvate; TCA: Tri-carboxylic acid; PTS: Phosphotransferase System; UTase: Uridylyltransferase.

## Competing interests

The authors declare that they have no competing interests.

## Authors' contributions

LWM carried out fermentation experiments, assayed, made statistical analysis, and drafted the manuscript. KS considered the experimental design, analyzed the result, and prepared the manuscript together with LWM. All authors read and approved the final manuscript.

## Supplementary Material

Additional file 1 **Effect of phosphate concentration on the fermentation characteristics of wild type *E. coli *(a) and its *phoB *mutant (b)**. Click here for file

Additional file 2 **Global regulators and their regulated genes**. Click here for file

Additional file 3 **List of additional primers**. Click here for file
