# Supplementary material for: Metabolic regulation of Escherichia coli and its phoB and phoR genes knockout mutants under phosphate and nitrogen limitations as well as at acidic condition
Source: Microb Cell Fact. 2011 May 20;10:39. doi: 10.1186/1475-2859-10-39 (PMC3129296; doi:10.1186/1475-2859-10-39)
Supplement: Additional file 1 — Effect of phosphate concentration on the fermentation characteristics of wild type E. coli (a) and its phoB mutant (b). [file 1475-2859-10-39-S1.PDF]

Additional file 1: Effect of phosphate concentrations on the fermentation characteristics of wild type *E.coli* (a) and its *phoB* mutant (b).

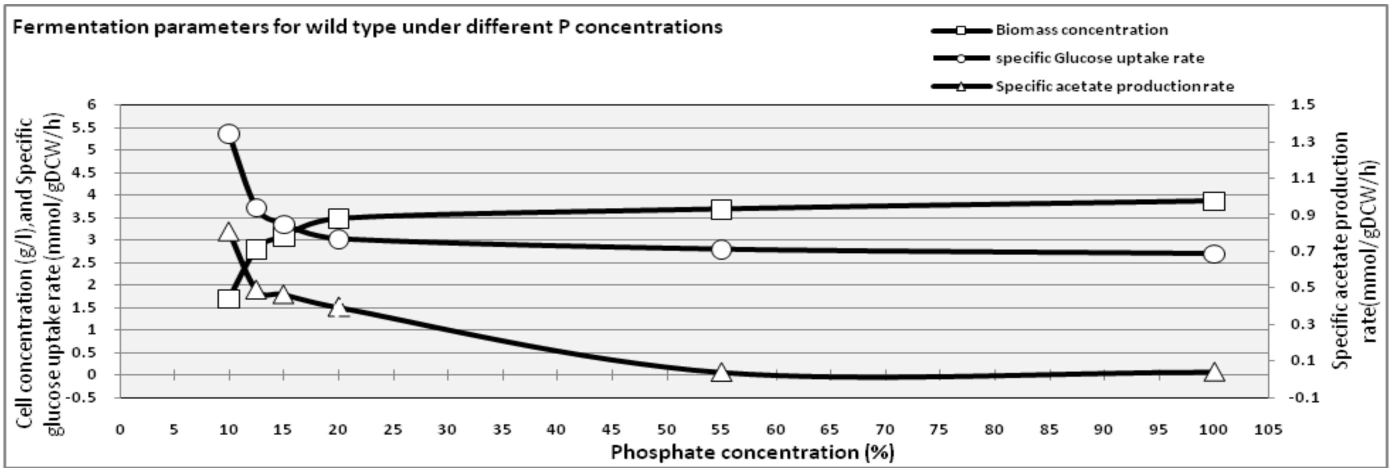

(a)

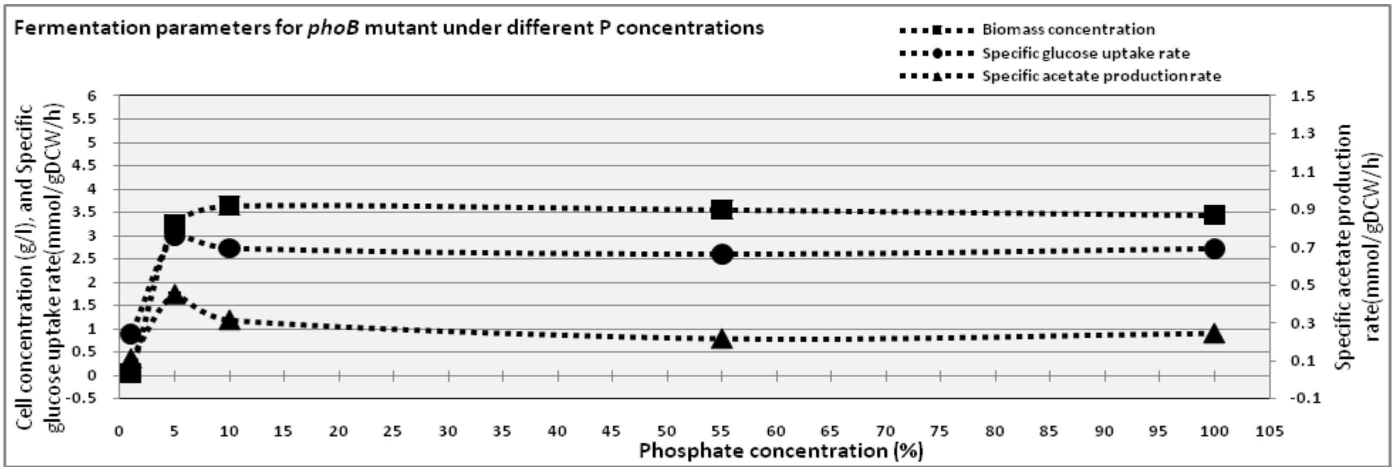

(b)
