# Supplementary material for: Metabolic regulation of Escherichia coli and its phoB and phoR genes knockout mutants under phosphate and nitrogen limitations as well as at acidic condition
Source: Microb Cell Fact. 2011 May 20;10:39. doi: 10.1186/1475-2859-10-39 (PMC3129296; doi:10.1186/1475-2859-10-39)
Supplement: Additional file 2 — Global regulators and their regulated genes. [file 1475-2859-10-39-S2.PDF]

Additional file 2: Global regulators and their regulated genes

ArcA/B:

+ : *pfl, cydAB*
_ : *aceBAK, aceEF, acnA, fumAC, gltA, icdA, lpdA, mdh, ptsG, sdhCDAB, cyo*

pdhR:

+ : *aceEF, ndh, yfiD*
_ : *cyoABCD*

Fur:

+ : *hmp*
_ : *cyoABCD, ompF*

Cra:

+ : *aceA, acnA, fbp, icdA, pckA, ppsA, cydB*
_ : *acnB, eda, edd, eno, gapA, pfkA, ptsHI, pykF*

Crp/Cya:

+ : *mlc, aceEF, acnAB, crr, fumA, gltA, mdh, pckA, ptsG, ptsHI, sdhABCD, sucABCD, tpiA, ompF, rpoS*
_ : *lpdA, aceBAK, acnA, cyoA, gdhA, glnAL, gltA, mdh, sdhCDAB, sodA, sucABCD, ugpA,*

Fnr:

+ : *frd, pfl, ackA, ndh, nuoA, pstSCAB-phoU, yfiD*
_ : *acnA, fumAC, icdA, lpdA, ptsG, sdhCDAB, talA, cyoABCD, cydAB*

PhoB:

+ : *phoBR, phoA-psiF, asr, pstSCAB-phoU*
_ : *phoH, phnCHN, ugpA, argP*

RpoS:

+ : *gadA, gadB, osmB, sodC, talA, tktB, acs, poxB, acnA, fumC*

SoxR/S:

+ : *sodA, zwf, rpoD, rpoS, fumC, tolC, micF, marA*
_ : *rob*

Mlc

+ : *ldhA*
_ : *crr, ptsG, ptsH1, manXYZ, malT*

GadE

+ : *gadE, gadXW*
_ : *cyoABCDE, gltB*
