# Supplementary material for: Metabolic regulation of Escherichia coli and its phoB and phoR genes knockout mutants under phosphate and nitrogen limitations as well as at acidic condition
Source: Microb Cell Fact. 2011 May 20;10:39. doi: 10.1186/1475-2859-10-39 (PMC3129296; doi:10.1186/1475-2859-10-39)
Supplement: Additional file 3 — List of additional primers. [file 1475-2859-10-39-S3.PDF]

**Additional file 3:** List of additional primers

| | |
|---|---|
| *phoB***:** | Left primer: TTAAACCACGTCTGGGGAAC<br>Right primer: TAAAAGCGGGTTGAAAAACG |
| *phoA:* | Left primer: ACGAAAAAGATCACCCAACG<br>Right primer: GATCCTTTTCCGCCTTTTTC |
| *phoE:* | Left primer: TGGGGCCTATACCAACTCAG<br>Right primer: GCCAGTTATTGGCGTCATTT |
| *phoH:* | Left primer: AAGGAAGCCAACCCTCTGAT<br>Right primer: GGCCATACCAATGGCTTCTA |
| *phnC:* | Left primer: CGTACTGGAGAACGTGCTGA<br>Right primer: GTTCATCGGCCAGAATCACT |
| *pstS:* | Left primer: AGCTACCTGGCGAAAGTGAA<br>Right primer: GGTGTACGCCAGGTTGTTCT |
| *ugpB:* | Left primer: CAAAGCAGCGTATGACCTGA<br>Right primer: GGTGTCTTCTTACCGGTCCA |
| *phoM* (*creC*)*:* | Left primer: CTGGATAACGCCATCGATTT<br>Right primer: ATTTGCACGAGGCAAAGAGT |
| *phoR:* | Left primer: TGGAATTTATTGCGCCTTTC<br>Right primer: ACCAGAAAATACCGCCCTCT |
| *phoU:* | Left primer: CGTCAACATGATGGAAGTGG<br>Right primer: CCAGCGACTCCAGACTTACC |
| *asr:* | Left primer: CGCTGCTATGGGTCTGTCTT<br>Right primer: TTTTGTTCAGGGGCTTTCTG |
